# Supplementary material for: Modeling glioblastoma heterogeneity as a dynamic network of cell states
Source: Mol Syst Biol. 2021 Sep 16;17(9):e10105. doi: 10.15252/msb.202010105 (PMC8444284; doi:10.15252/msb.202010105)
Supplement: Supplementary file 6 — Source Data for Figure 5 [file MSB-17-e10105-s004.zip › Figure5A_sourcedata/GSEA_3017/nosigdb_stateB.GseaPreranked.1622795021725/NB_KI_CLUSTER6.html]

Details for gene set NB\_KI\_CLUSTER6[GSEA]

|  || Dataset | state43017 |
| Phenotype | NoPhenotypeAvailable |
| Upregulated in class | na\_neg |
| GeneSet | NB\_KI\_CLUSTER6 |
| Enrichment Score (ES) | -0.3688677 |
| Normalized Enrichment Score (NES) | -1.9467207 |
| Nominal p-value | 0.004854369 |
| FDR q-value | 0.012892825 |
| FWER p-Value | 0.051 |
Table: GSEA Results Summary

  

Fig 1: Enrichment plot: NB\_KI\_CLUSTER6      
 Profile of the Running ES Score & Positions of GeneSet Members on the Rank Ordered List

  

| PROBE | GENE SYMBOL | GENE\_TITLE | RANK IN GENE LIST | RANK METRIC SCORE | RUNNING ES | CORE ENRICHMENT || 1 | UPP1 |  |  | 74 | 0.572 | -0.0018 | No |
| 2 | DLG1 |  |  | 347 | 0.327 | -0.3128 | Yes |
| 3 | SOGA1 |  |  | 378 | 0.314 | -0.2994 | Yes |
| 4 | ZNF714 |  |  | 382 | 0.313 | -0.2497 | Yes |
| 5 | TTYH3 |  |  | 396 | 0.309 | -0.2141 | Yes |
| 6 | NUP107 |  |  | 430 | 0.298 | -0.2076 | Yes |
| 7 | G2E3 |  |  | 446 | 0.294 | -0.1775 | Yes |
| 8 | TERF1 |  |  | 451 | 0.293 | -0.1326 | Yes |
| 9 | P4HTM |  |  | 452 | 0.292 | -0.0826 | Yes |
| 10 | HSPA4L |  |  | 461 | 0.290 | -0.0437 | Yes |
| 11 | TBC1D5 |  |  | 494 | 0.282 | -0.0385 | Yes |
| 12 | TMTC3 |  |  | 569 | 0.269 | -0.0922 | Yes |
| 13 | CEP44 |  |  | 643 | 0.256 | -0.1467 | Yes |
| 14 | TSPAN5 |  |  | 646 | 0.256 | -0.1055 | Yes |
| 15 | FAM76B |  |  | 653 | 0.255 | -0.0697 | Yes |
| 16 | CBY1 |  |  | 666 | 0.253 | -0.0426 | Yes |
| 17 | MSMO1 |  |  | 691 | -0.267 | -0.0292 | Yes |
| 18 | CHD7 |  |  | 706 | -0.284 | 0.0007 | Yes |
| 19 | SQLE |  |  | 735 | -0.405 | 0.0324 | Yes |
Table: GSEA details [plain text format]

  

Fig 2: NB\_KI\_CLUSTER6: Random ES distribution      
 Gene set null distribution of ES for **NB\_KI\_CLUSTER6**
